# TDP-43 and Inflammation: Implications for Amyotrophic Lateral Sclerosis and Frontotemporal Dementia

**DOI:** 10.3390/ijms22157781

**Published:** 2021-07-21

**Authors:** Fiona Bright, Gabriella Chan, Annika van Hummel, Lars M. Ittner, Yazi D. Ke

**Affiliations:** Dementia Research Centre, Department of Biomedical Science, Faculty of Medicine Health and Human Sciences, Macquarie University, Sydney, NSW 2109, Australia; gabriella.chan1@hdr.mq.edu.au (G.C.); annika.vanhummel@mq.edu.au (A.v.H.); lars.ittner@mq.edu.au (L.M.I.)

**Keywords:** TDP-43, neuroinflammation, neurodegeneration, immunity, ALS, FTD

## Abstract

The abnormal mislocalisation and ubiquitinated protein aggregation of the TAR DNA binding protein 43 (TDP-43) within the cytoplasm of neurons and glia in the central nervous system (CNS) is a pathological hallmark of early-onset neurodegenerative disorders amyotrophic lateral sclerosis (ALS) and frontotemporal dementia (FTD). The pathomechanisms underlying abnormal mislocalisation and aggregation of TDP-43 remain unknown. However, there is a growing body of evidence implicating neuroinflammation and immune-mediated mechanisms in the pathogenesis of neurodegeneration. Importantly, most of the evidence for an active role of immunity and inflammation in the pathogenesis of ALS and FTD relates specifically to TDP-43, posing the question as to whether immune-mediated mechanisms could hold the key to understanding TDP-43’s underlying role in neurodegeneration in both diseases. Therefore, this review aims to piece together key lines of evidence for the specific association of TDP-43 with key immune and inflammatory pathways to explore the nature of this relationship and the implications for potential pathomechanisms underlying neurodegeneration in ALS and FTD.

## 1. Introduction

### 1.1. Neuroinflammation

The innate immune system is the body’s first line of defence against pathogens and plays a central yet varying role in both health and disease. Neuroinflammation involves a complex multistage physiological response triggered by cell damaging processes in the brain, including infection, toxins, autoimmunity, trauma, and aberrant responses to altered neuronal activity. Neuroinflammation is driven by reactive resident CNS innate immune glial cells, microglia, and astrocytes, and is accompanied by a dynamic biochemical cascade of inflammatory cytokines and chemokines that modify the CNS microenvironment. The degree of neuroinflammation that exists across the spectrum of neurological conditions varies and is dependent on multiple factors including the duration of the inflammatory response, its course and the circumstances underlying the primary insult. The primary aim of the neuroinflammatory response is to mitigate the triggering factors by invoking CNS immunity to defend from harm and maintain and restore homeostasis. Therefore, neuroinflammation has the capacity to be both beneficial and damaging [1,2].

The brain has been shown to undergo a process termed ‘inflammaging’, progressively acquiring an increased proinflammatory environment across the lifespan [3]. In addition to a heightened inflammatory environment with ageing, the immune system itself undergoes a gradual deterioration or remodelling termed ‘immunosenescence’. This is responsible for the increased susceptibility of aged individuals to diseases, particularly inflammatory age-related conditions. Furthermore, advancing age is a primary risk factor for neurodegenerative diseases as recently reviewed [4]. Despite neurodegenerative diseases exhibiting different aetiologies, neuroinflammation is considered a characteristic pathological feature across their spectrum [5,6,7,8] and in other neurological conditions including stroke, traumatic brain injury (TBI), chronic traumatic encephalopathy (CTE), and neurological cancers. However, the question remains as to whether neuroinflammation is a primary or secondary insult in the pathogenesis of neurodegeneration. Determining the extent of the role of neuroinflammation in neurodegenerative disease pathogenesis has the potential to provide a tool for biomarker and drug discovery across the spectrum of clinical neurodegenerative disease subtypes, this is a highly active area of research.

### 1.2. TAR DNA Binding Protein 43 (TDP-43)

**Figure 1:**
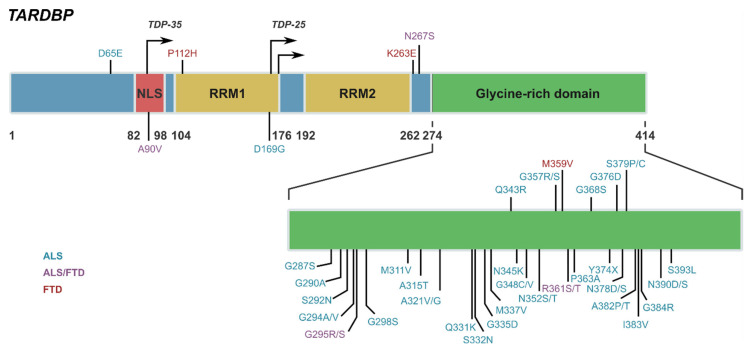
Structure of TDP-43. TDP-43 is a 43 kDa, 414 amino acid nuclear RNA/DNA-binding encoded by the *TARDBP* gene and part of the heterogeneous nuclear ribonucleoprotein (hnRNP) family. Structurally TDP-43 consists of an N-terminal and two DNA/RNA binding domains (RRM1 and RRM2) followed by a glycine rich C-terminal domain where TDP-43 mediates protein–protein interactions and where the majority of all pathogenic *TARDBP* mutations have been identified to date for both ALS and FTD. Predominantly localised to the nucleus owing to its nuclear localisation signal (NLS), TDP-43 shuttles between the nucleus and cytoplasm via nucleocytoplasmic transport, assisting with the regulation of many aspects of RNA processing including splicing, trafficking, stabilisation, and miRNA production. Structural, post-translational modifications to TDP-43 can occur during pathological conditions, causing abnormal mislocalisation and accumulation in the cytoplasm of neurons and proteolytic cleavage of TDP-43 into abnormal C-terminal fragments, TDP-35 and TDP-25. Alterations in the RRM1 of TDP-43 via oxidation can induce its aggregation and mislocalisation into the cytoplasm and misfolding of the RRM1 domain is thought to underlie TDP-43 misfolding, oligomerisation, accumulation, and ultimately proteinopathy. Both the NLS and RRM1 domain of TDP-43 are potential structural sites playing an important facilitatory role in the interaction of TDP-43 with central inflammatory pathways including NF-κβ/p65.

TDP-43 is a 43 kDa, 414 amino acid nuclear RNA/DNA-binding protein that is widely expressed, but of functional relevance to the CNS. TDP-43 is encoded by the *TARDBP* gene and is part of the heterogeneous nuclear ribonucleoprotein (hnRNP) family. TDP-43 was first identified as a host cell protein binding to pyrimidine-rich DNA motifs in a long terminal repeat, referred to as a TAR of human immunodeficiency virus type I [9]. Structurally, TDP-43 consists of an N-terminal and two DNA/RNA binding domains (RRM1 and RRM2) followed by a glycine rich C-terminal where TDP-43 mediates protein–protein interactions and where the majority of all pathogenic *TARDBP* mutations have been identified to date [10,11] (Figure 1). TDP-43 is consistently reported to bind to UG rich domains within proximal intron regions of 3′ untranslated regions of mRNA encoding proteins. While the complete function of TDP-43 remains to be determined, under normal physiological conditions TDP-43 exhibits a variety of important functions involved in RNA biogenesis and processing [12,13]. Predominantly localised to the nucleus owing to its nuclear localisation signal (NLS), TDP-43 has the capacity to shuttle between the nucleus and cytoplasm via nucleocytoplasmic transport, assisting with the regulation of various aspects of RNA processing including splicing, trafficking, stabilisation, and miRNA production [12,14,15]. TDP-43 was previously identified to also exhibit a nuclear export sequence (NES) to facilitate cytoplasmic shuttling out of the nucleus, however recent evidence has challenged this, demonstrating that TDP-43 diffusively passes between the nucleus and cytoplasm and the NES is not required for export [16,17,18]. TDP-43 is reportedly enriched in dendrites of neurons where it plays a role in mRNA transport and local translation in dendritic spines [19,20]. TDP-43 is self-regulatory, tightly controlling its own transcription via negative feedback mechanisms and promoting degradation of *TARDBP* transcripts [21]. This function is essential given that overexpression or deletion is detrimental to TDP-43 survival [22,23,24]. TDP-43 has been shown to interfere with lysosomal function and therefore its own degradation via lysosomal pathways and trigger lethal autophagy [25].

Under pathological conditions, structural post-translational modifications to TDP-43 can occur causing abnormal mislocalisation and accumulation in the cytoplasm of neurons and proteolytic cleavage of TDP-43 into abnormal C-terminal fragments. Notably, alterations in the RNA recognition domain, RRM1 of TDP-43 via oxidation can induce its aggregation and mislocalisation into the cytoplasm [26,27,28] and structure/function analysis has shown that misfolding of the RRM1 domain could underlie TDP-43 misfolding, oligomerisation, accumulation, and ultimately proteinopathy [27,29]. TDP-43 is identified as a major disease associated protein in early-onset neurodegenerative diseases amyotrophic lateral sclerosis (ALS) and frontotemporal dementia (FTD). In neurodegeneration, TDP-43 undergoes various post translational modifications including ubiquitination, phosphorylation, and acetylation, all of which alter its structure and function [30,31]. The phenomenon of these alterations remains unexplained, with debate as to whether such modifications are secondary or disease causative. However, the partially helical region in the disordered C-terminal domain harbours multiple mutations associated with ALS and FTD and is important for TDP43 function and liquid–liquid phase separation. Furthermore, pathological TDP-43 aggregates that accumulate within the brain of ALS and FTD patients contain proteolytically cleaved C-terminal fragments, TDP-35 (35 kDa) and TDP-23 (25 kDa), which must be cleared from cells to prevent further aggregation and sequestration of neuronal components that result in toxicity [32]. The pathophysiology of these TDP-43 C-terminal fragments or CTFs is yet to be determined; however it is an active area of research [33]. Ubiquitinated protein aggregates of TDP-43 are present in neurons and glia of the brain in 95% of ALS patients and in approximately 50% of FTD patients [34,35], and regional pathology of TDP-43 is associated with distinct clinical phenotypes of disease [36]. TDP-43 can be detected in the CSF, with ALS and FTD patients identified to have increased levels of TDP-43 in the CSF compared to age-matched controls [37,38,39]. While primarily a pathological feature in ALS and FTD, the abnormal deposition of TDP-43 has also been reported across the spectrum of neurodegenerative diseases including Alzheimer’s disease (AD) [40], Parkinson’s disease (PD) [41] Huntington’s disease [42] and CTE [43]. TDP-43 proteinopathy has also been reported within pathologic astrocytes in the brain of patients with the rare neurodegenerative Alexander disease [44] and in the autosomal recessive lysosomal storage disorder Nieman Pick disease [45].

The pathomechanisms resulting in abnormal mislocalisation and aggregation of TDP-43 within the CNS in ALS and FTD remain unknown, however there is a growing body of evidence to support an important role of neuroinflammation and innate immune-mediated mechanisms underlying the pathogenesis of neurodegeneration as recently reviewed for both diseases respectively [6,7]. However, to date most of the evidence for an active role of innate immunity in the pathogenesis of ALS and FTD related specifically to TDP-43 has yet to be drawn together to fully explore the relationship between TDP-43 and immune-mediated pathways. To explore this further, this review will piece together published evidence to date and establish four key lines of evidence supporting a link between TDP-43, immunity, and inflammation. It is anticipated that this review will serve as a platform for future research that aims to explore the molecular mechanisms underlying TDP-43’s relationship with immunity and inflammation in the pathogenesis of neurodegeneration in ALS and FTD, potentially providing novel therapeutic targets.

## 2. ALS and FTD Causative and Susceptibility Genes Associated with TDP-43 Implicated in Immunity and Inflammation

A significant line of evidence for the role of immunity and inflammation in the pathogenesis of neurodegeneration underlying ALS and FTD is that the majority of causative and susceptibility genes associated with TDP-43 pathology are highly expressed in innate immune cells and are increasingly implicated in key immune and inflammatory pathways. These genes include *C9orf72*, *GRN*, and *TBK1*, among others (Table 1). In the largest cohort of unrelated patients with FTD-TDP to date, Pottier and colleagues performed a comprehensive genome wide association study (GWAS) and identified significant genomic loci within the human leukocyte antigen (HLA) locus (HLA-DQAZ, cell-surface proteins responsible for the regulation of the immune system) in addition to a rare loss of function variants in genes involved in the *TBK1*-immunity pathway [46]. These findings strongly implicate immune pathways in the pathogenesis of FTLD-TDP specifically, providing further evidence for immune dysregulation in the pathogenesis of FTD [46]. In support of this, also using GWAS, Broce and colleagues demonstrated an immune-mediated genetic enrichment in the HLA region specifically and showed novel candidate FTD susceptibility loci in *TBK1*. The investigators suggested that for a subset of FTD patients, immune dysfunction may contribute to increased FTD risk [47]. While not investigated in the study, this subset could reflect that of FTD-TDP.

## 3. Chromosome Open Reading Frame 72 (C9orf72)

The most common gene abnormality in both ALS and FTD is the presence of expanded hexanucleotide repeat sequences in the noncoding region of the *C9orf72* gene [80,81]. *C9orf72* repeat expansions produce TDP-43 post-mortem pathology in both ALS and FTD [80,81,82]. Although the function of *C9orf72* remains unknown, *C9orf72* knockout rodent studies demonstrate a systemic proinflammatory state, severe autoimmune disease [50,51,52,83,84], mild neuroinflammation characterised by increased expression of IL6 and IL1β in microglia, and an upregulation of inflammatory genes in the spinal cord compared to control mice [85]. *C9orf72* expansions in innate immune cells result in the loss of function toxicity by impairment of cellular homeostatic processes such as autophagy [86]. Of note, *C9orf72* expression is higher in microglia than in any other cell type and is also expressed highly in dendritic immune cells, indicating that *C9orf72* enacts a central role in the maintenance of immune homeostasis [85]. Post-mortem brain analysis of white matter regions in the motor cortex shows a greater microglial presence in individuals with *C9orf72* mediated ALS compared to sporadic ALS [87], indicating a heightened innate immune response linked to *C9orf72* repeat expansion. Furthermore, loss of *C9orf72* function may have consequences for microglial function in clearing aggregated proteins, which may cause persistent microglial activation that further exacerbate the progression and development of ALS and FTD [85].

## 4. Granulin (GRN)

*GRN* encodes the secreted protein progranulin (PGRN). In the CNS, PGRN plays a critical role in maintaining physiological functions and is expressed by neurons and microglia within the CNS [88,89]. PGRN has a major role in regulating lysosomes and microglial responses and acts as a chemoattractant for microglia [90,91,92]. Haploinsufficiency caused by an autosomal dominant mutation within *GRN* is a major cause of familial FTD [89,93]. *GRN* mutations are associated with the accumulation of abnormal TDP-43 pathology [34] and symptomatic *GRN* carriers present with dysregulated levels of proinflammatory cytokines in serum and CSF, in addition to increased expression of inflammatory genes in leukocytes [94]. *GRN* knockout mice with PGRN deficiency exhibit an excessive accumulation of activated microglia [95] that produce an excess of proinflammatory cytokines rendering them neurotoxic [96,97]. Emerging evidence also indicates that PGRN deficiency promotes lysosomal dysfunction and production of complement factors that preferentially affect synaptic connections in the thalamocortical circuit [98]. In support of this, *GRN* knockout mice present with a chronic upregulation of innate immunity and complement expression that increases with age [99]. It has been proposed that haploinsufficiency of PGRN could contribute to pathomechanisms underlying FTD via lysosomal dysfunction and neuroinflammation [98].

## 5. TANK Binding Kinase 1 (TBK1)

*TBK1* encodes a protein kinase with an established role in regulating immune response, autophagy, and neuroinflammation [55,100]. In innate immune signalling, *TBK1* is activated via multiple pathways resulting in phosphorylation and activation of innate immune transcription factors, interferon regulatory factor 3 and 7 (IRF3/IRF7), and activates NF-κB in response to TNFα. Mutations including loss of function, missense, and in-frame deletions of *TBK1* are identified as causative of ALS and FTD [101,102]. Post-mortem neuropathological analysis of the *TBK1* mutation carriers show TDP-43-positive perinuclear inclusions in temporal lobe neurons, but not in the spinal cord [103]. As discussed above, GWAS studies have reported an excess of rare loss-of-function variants in the *TBK1*-related innate immunity pathway in FTD-TDP patients compared to controls [46] and *TBK1* mutations observed in ALS and FTD-TDP patients have previously been shown to reduce the activation of one of the most well-characterised transcription factors involved in innate immunity, interferon regulatory factor 3 (IRF3) [55]. *TBK1* mutations could influence the pathogenesis of ALS and FTD given the role of *TBK1* in autophagy processes. In support of this, mutations in the autophagy receptors *OPTN* and *SQSTM1* are causal of FTD and ALS and *TBK1* promotes autophagy via phosphorylation of *OPTN* and *SQSTM1* therefore enhancing the ubiquitin-binding abilities of both proteins [104,105]. *TBK1* could potentially represent a direct link between neuroinflammation and kinases in the neurodegeneration underlying ALS and FTD.

## 6. Relationship between TDP-43 and Key Innate Immune Inflammatory Pathways

### 6.1. TDP-43 and NF-κβ/p65

One of the most prolific inflammatory pathways consistently associated with TDP-43 to date is the nuclear factor kappa light chain enhancer of activated B cells NF-κβ. NF-κβ acts as a transcription activator and modulates hundreds of genes involved in inflammation, innate immunity, cell survival, and cancer. This pathway is identified as a molecular culprit of inflammaging [106], is involved in neuroinflammatory conditions including cerebral ischemia and TBI, and has been implicated in neurodegenerative diseases [107,108,109]. Activation via various cell surface factors translocate NF-κβ to the nucleus, which can be measured by the nuclear presence of its most abundant subunit, p65. The NF-κβ/p65 subunit (also known as RelA), centrally regulates innate immunity [110]. TDP-43’s involvement in NF-κβ pathways has been reported in both neurons and microglia [111,112]. While this review focuses specifically on the relationship between TDP-43 and NF-κβ pathways, it is important to note that activation of NF-κβ pathways has also been demonstrated in SOD1 ALS mouse models [113,114].

Of particular interest to this review, TDP-43 and NF-κβ share the same classic nuclear transportation mechanism, therefore it is suggested that they may functionally interact with each other by competing for access to nuclear transportation machinery [115]. In support of this, TDP-43 has been shown to regulate NF-κβ signalling. Using various cell culture and transfection approaches, Zhu and colleagues demonstrated that overexpression of TDP-43 constituently inhibits the NF-κβ pathway. This inhibition was attributed to the competitive binding of TDP-43 to the nuclear translocation importin a3 (KPNA4) via TDP-43’s NLS. This was supported by multiple lines of evidence including the observation that a mutant TDP-43 lacking an NLS was unable to inhibit the inflammatory cytokine TNFα-induced p65 nuclear translocation in a dose dependent manner. Furthermore, silencing TDP-43 using siRNA increased p65 nuclear localisation upon TNFα stimulation, suggesting that p65 nuclear translocation is actively inhibited by TDP-43 [115]. Ultimately TDP-43 may act as a default suppressor of the NF-κβ transactivation pathway. Conversely, the blockage of NF-κβ nuclear translocation by overexpression of TDP-43 is preventable by simultaneous overexpression of p65 [115]. In a previous study by Swarup and colleagues, while it was demonstrated that TDP-43 interacts and serves as a coactivator of NF-κβ in cultured cells including neurons and glia, contrastingly it was demonstrated that TDP-43 itself does not activate NF-kβ or upregulate p65 [116]. Rather a second hit or inflammatory trigger (e.g., LPS, PAMPs, or cytokines) is required to cause NF-kβ activation via TLR signalling. In conjunction with this second hit, TDP-43 overexpression can enhance NF-kβ activation and the deregulation of TDP-43 may contribute to ALS pathogenesis in part by this enhancement [116].

In addition to the role TDP-43’s NLS may play in the interaction with NF-κβ/p65, other key structures of TDP-43 such as the RRM1 domain may also play a facilitatory role. The sensitivity of the RRM1 domain in TDP-43 proteinopathy has been highlighted and oxidation of the RRM1 domain results in cytosolic mislocalisation with irreversible protein aggregation [26,27]. Interestingly, aside from RNA metabolism, the RRM1 domain itself is responsible for the interaction between TDP-43 and p65 [116]. This interaction mediates overactivation of the NF-κβ pathway and results in a heightened vulnerability of neurons to injury and a hyperactive inflammatory response of glial cells [116]. The abnormal binding of p65 to the RRM1 domain of TDP-43 is also proposed to interfere with normal protein folding or RNA binding, resulting in TDP-43 aggregation in the cytoplasm [114].

To therapeutically reduce TDP-43 pathology, Pozzi and colleagues utilised the implicated role of the RRM1 domain in an interaction with the NF-κβ/p65 subunit to generate single chain antibodies targeting the RRM1 domain. The twofold aim was to block TDP-43 and p65 interaction, thus reducing NF-κβ activation and interfering with TDP-43 protein aggregation [117]. Virus mediated delivery of this novel single chain antibody against TDP-43 (VH7VK9) in the CNS of transgenic mice expressing mutant hTDP-43 successfully improved cognitive and motor deficits in addition to decreasing TDP-43 proteinopathy, neuroinflammatory changes, and NF-κβ activation in microglial cells [117]. This suggests that NF-κβ inhibition may restore TDP-43 function. Alternatively, NF-κβ could contribute to the clearance of TDP-43 within the neuronal cytoplasm via protein degradation pathways such as ubiquitin–proteosome and autophagy processes [114,118].

In a separate study, transgenic mice with neuron specific expression of the super-repressor form of NF-κβ (IkBa-SR) were crossed with mice of both sexes expressing ALS-linked gene mutations for TDP-43. Neuronal expression of IkBa-SR in mice expressing TDP43^A315T^ or TDP43^G348C^ resulted in a decrease in the ratio of cytoplasmic to nuclear expression of human TDP-43, partial rescue of large spinal motor neurons at one year of age and improved motor performance and cognition. Neuronal inhibition of NF-κβ therefore rescued TDP-43 proteinopathy and mitigated TDP-43 neurodegeneration [114]. These observations aligned with the conditional suppression of mutant TDP-43, which resulted in improved cognition demonstrated in a previous study using TDP-43^A315T^ mice [119].

In the context of neurodegenerative disease TDP-43 has been shown to directly interact with p65 colocalising in the nucleus of neurons in CNS samples from ALS patients [116], patients with mild cognitive impairment (MCI) and episodic memory deficits [120], and in transgenic mice that overexpress human wild type and mutant TDP-43 [116]. Levels of mRNA and protein of both TDP-43 and NF-κβ are higher in the spinal cord of ALS patients than control individuals [111,116] and various links between ALS and NF-kβ have recently been reviewed elsewhere [121]. Investigation into the potential effects of NF-κβ activation by inflammatory stimuli on TDP-43 redistribution in various cultured cells (i.e., microglia, astrocytes, and neurons) chronic brain inflammation induced by stimuli of NF-κβ signalling such as TNFα or LPS was found to mediate TDP-43 proteinopathy [111]. This was further supported by in vivo investigation in mice expressing human TDP-43^A315T^, where chronic administration of LPS from 6 months of age exacerbated pathological TDP-43 accumulation in the cytoplasm of spinal motor neurons and enhanced levels of TDP-43 aggregation [111].

It is clear from multiple studies that a functional interaction between NF-κβ/p65 and TDP-43 exists, although it is yet to be fully understood, there is evidence to suggest such an interaction may be mediated by microglia. In addition to being a coactivator of p65, overexpression of TDP-43 has been shown to produce a hyperactive proinflammatory response after stimulation with LPS and ROS, resulting in microglial sensitivity to immune stimulation, subsequently enhancing the neurotoxicity of neighbouring neurons [116]. Downregulation of TDP-43 has been demonstrated to reduce activation of NF-κβ and TDP-43 has also been shown to activate microglia via the NF-κβ signalling pathway and the NLRP3 inflammasome [112]. In cell culture, wild type truncated 25kD C-terminal fragments and mutant forms of TDP-43 activate microglia, upregulating inflammatory factors NOX2, TNFα, and IL1β, however wild type forms are significantly less effective in activating microglia compared to mutant. The observed response to TDP-43 was mediated by its interaction with the microglial surface receptor CD14, which stimulated the NF-κβ pathway in addition to the intracellular inflammasome [112]. Blockage at the cell surface using CD14 blocking antibodies suppressed microglial NF-κβ activation and proinflammatory cytokine production mediated by TDP-43. In culture, the addition of the mutant TDP-25^A315T^ fragment to motoneurons alone or to motoneurons cocultured with microglia mediated activation of microglia and triggered a proinflammatory cascade that was toxic to motoneurons. However, in the absence of microglia, the TDP-25^A315T^ fragment was not toxic to motoneurons, suggesting that TDP-43 neurotoxicity is indirect and mediated via proinflammatory microglia [112].

### 6.2. TDP-43 and cGAS/STING Pathway

The recognition of foreign nucleic acids is one of the key mechanisms utilised by the immune system to detect pathogenic entities to elicit a response. A signal is relayed via the cytoplasmic DNA-sensing cyclic GMP-AMP synthase (cGAS)/stimulator of the interferon genes (STING) pathway (cGAS/STING) following detection of cytosolic DNA, which then induces an immune response. Increased engagement of the cGAS/STING pathway in the CNS results in neuroinflammation and neurodegeneration as recently reviewed [122]. STING can drive the activation of NF-κβ and type I IFN pathways, which are each elevated in ALS and is suggested to contribute to progression of TDP-43 driven neurodegeneration [123]. ALS-associated mutations have been shown to enhance the accumulation of TDP-43 within mitochondria [124,125], this could represent the specific way in which TDP-43 alters homeostasis of the cell and could be consequential in triggering an immune response [123]. Recently it was reported that TDP-43 causes inflammation by stimulating mitochondrial DNA release, which is subsequently sensed by the cytosolic cGAS/STING pathway. In a comprehensive study [123] using induced pluripotent stem cell (iPSC)-derived motor neurons and TDP-43 mutant mice, it was demonstrated that TDP-43 causes inflammation in ALS by triggering the release of mitochondrial DNA into the cytoplasm, subsequently activating the cGAS/STING pathway, and resulting in neuroinflammation. Prior to this study no immune sensor had been identified to detect cytoplasmic TDP-43 and trigger the inflammatory response observed in TDP-43 proteinopathies, therefore, providing insight into how neuroinflammation is potentially triggered in TDP-43 proteinopathies is fundamentally important in understanding disease mechanisms. Interestingly, the adaptor protein downstream of STING is *TBK1*, and as discussed above the *TBK1* immune pathway has been implicated in both FTD and ALS [46,47].

### 6.3. TDP43 and NLRP3 Inflammasome

The NLR family pyrin domain containing 3 (NLRP3) inflammasome is a member of a family of intracellular innate immune sensors that are integral for cellular defence. NLRP3 is activated by pathogen associated messenger proteins (PAMPs) and danger associated messenger proteins (DAMPs) that signal and activate microglia and are involved in the response to glial production of various inflammatory factors, subsequently promoting an inflammatory response that further engages the innate immune system [126]. NLRP3 is critical for production of proinflammatory cytokines IL-1β and IL-18 and is therefore a key target for modulation of the initiation and progression of neuroinflammation [127]. Increasing evidence implicates the NLRP3 inflammasome in multiple neurodegenerative disorders [128].

TDP-43 inclusions activate the NLRP3 inflammasome in primary microglial cultures resulting in increased production of IL-1β [112]. Similar increases in expression of NLRP3 are also observed in post-mortem tissue from individuals with sporadic ALS [129]. NLRP3 activation has been shown to play a distinct role in the upregulation of nuclear TDP-43 and TDP-43-induced neurotoxicity by downregulation of the cytosolic E3 ubiquitin ligase *Parkin* in the hippocampus of mice treated with the neurological toxicant BDE-47 [130]. *Parkin* plays an important role in mitochondrial activity and integrity and has been shown to link together inflammation, mitochondrial stress, and neurodegeneration with an additional role in restraining innate immunity [131]. Although mutations in *Parkin* are predominantly linked to PD there is increasing evidence that *Parkin* facilitates TDP-43 translocation from the nucleus to the cytoplasm and has an essential role in TDP-43 subcellular localisation and toxicity [132,133]. In spinal cord samples from sporadic ALS patients, neurons with TDP-43 inclusions have decreased *Parkin* protein levels [134]. Given NLRP3 acts as a molecular platform for activation of caspase-1, which is shown to mediate *Parkin* cleavage, it has been hypothesised that NLRP3 inflammasome activation may be associated with TDP-43 toxicity [130]. *Drosophila* studies have suggested that the *Parkin* pathway may be differentially dysregulated in TDP-43 proteinopathy [135]. 

Deora and colleagues [136] demonstrated an upregulation of microglial NLRP3 in TDP-43^Q331k^ ALS mice, with TDP-43 wild-type and mutant proteins able to activate microglial inflammation in a NLRP3-dependent manner. Explicitly, spinal cord gene expression of the inflammasome components NLRP3, caspase 1, and ASC were significantly increased in TDP-43^Q331K^ mice compared to wild type littermates and both wild type and mutant (A315T and Q331K) recombinant forms of TDP-43 protein were able to activate primed microglia to generate the cytokine interleukin 1-beta (IL-1β). However, this was not unique to TDP-43 given aggravated and soluble SOD1^G93A^ also activates NLRP3 in primary mouse microglia. Of particular interest to ALS and FTD, NLRP3 inflammatory activation can be generated by *C90rf72* repeat expansions, leading to lysosomal dysfunction, mitochondrial functional impairments, intracellular metabolic imbalances, and intracellular protein aggregation [137].

### 6.4. TDP43 and MAPK Pathway

Stress activated protein kinases (SAPKs) are members of the mitogen activated protein kinase (MAPK) family and are activated by multiple environmental stressors including inflammatory cytokines. Several kinases including p38, JNK, and TBK1 have been associated with ALS-related pathophysiology [138,139]. Zhan et al. 2015 explored the relationship between TDP-43 and the cell stress response, focusing on the extensively investigated stress response pathway governed by SAPKs Jun N terminal kinase (JNK) and p38 mitogen activated protein kinase using a *Drosophila* model. JNK and p38 undertake multiple cellular functions within the CNS including prominent roles in both innate and adaptive immunity and the regulation of activity and expression of key inflammatory mediators as reviewed elsewhere [140].

In *Drosophila* models, neuroinflammation is demonstrated as a prominent feature of TDP-43 induced neurodegeneration and notably the innate immune response provides a strong phenotypic rescue in TDP-43 transgenic flies, dramatically extending their lifespan [141]. Both JNK and p38 are identified as immune response kinases responsible for regulating fly immunity and oxidative stress and the innate immune response are identified as key determinants of TDP-43 mediated toxicity in *Drosophila* motor neurons [141]. Zhan and colleagues demonstrated that p38 promotes oxidative stress and neuroinflammation whereas JNK antagonised oxidative stress and neuroinflammation, thus demonstrating the important yet opposing roles of these kinases in TDP-43-induced neurodegeneration [141]. Furthermore, key immune modulatory pathways in *Drosophila* including Toll/Dif and Imd/Relish have been shown to contribute to TDP-43 neurotoxicity, indicating that immune activation is a critical component of TDP-43 neurotoxicity in *Drosophila*. Given TDP-43 negatively regulates its own RNA [21], it has been suggested that cytosolic aggregation of TDP-43 may result in increased translation of feedforward TDP-43 aggregation and ultimately depletion of the essential TDP-43 splicing function within the nucleus [141]. This notion is further supported by the observation of nuclear clearing of TDP-43 in degenerating motor neurons of ALS and FTD patients [142].

Although TDP-43 has been shown to induce neuroinflammation, contradicting evidence investigating TDP-43 in the periphery demonstrated that overexpression of TDP-43 reduces the inflammatory response, interfering with the release of inflammatory factors. In a rat model of osteoarthritis (OA), the mechanism of TDP-43 gene expression on inflammatory factors JNK and p38 signalling pathways in ischemic hypoxic stress dependence was investigated. Overexpression of TDP-43 reduced the inflammatory response induced by OA by interfering with the release of inflammatory factors and inhibiting activation of the JNK and p38 signalling pathways via ischemic hypoxia stress [143]. These findings suggest that TDP-43 may alleviate the progression of OA to some extent, indicating a beneficial role for TDP-43 in the inflammatory response.

### 6.5. TDP-43 and Complement Cascade

The complement system is a branch of the innate immune system that plays a critical role in development, homeostasis, and regeneration of the CNS throughout life. This dynamic system consists of a group of proteins that work together to destroy foreign invaders, trigger inflammation, and remove debris from cells and tissues. Chronic activation of the complement system is a key mediator of neuroinflammation and complement dysregulation is identified as a key component in neurodegeneration. Notably the complement system has been proposed to drive neurodegenerative mechanisms in ALS [144,145]. In an ALS mouse model of TDP-43^Q331K^, local complement activation increased expression of C5aR1, part of the terminal complement pathway, which may contribute to motor neuron death and neuromuscular junction denervation. C5aR1 expression was upregulated during disease progression with expression in motor neurons and microglia surrounding regions of motor neuron death [146]. In addition, this model provided evidence for the dysregulation of aspects of each of the complement component pathways (i.e., classical, lectin, and alternative) in the spinal cord and tibialis anterior muscle during ALS disease progression [146]. This study demonstrated that complement activation and/or its dysregulation could play an important role in motor neuron loss and neuromuscular junction denervation and a heightened complement activation and enhanced C5aR1 signalling could contribute to the pathophysiology of the TDP-43^Q331K^ ALS model.

An important role for complement component proteins has been identified in tagging synapses during inflammation and remodelling [147]. Local translation of proteins at the synapse is an important aspect of neuronal function and the control of this translation involves silencing of translation by mRNA foci such as stress granules. TDP-43 has been identified to be expressed in these stress granules indicating that TDP-43 may to some extent be involved in the control of local synaptic translation [148,149]. The presence of TDP-43 at the synapse is likely important for the regulation of local protein translation and maintenance of normal synapses and TDP-43 may be involved in synaptic pruning with aging. Interestingly, in a knockout mouse model of fragile X syndrome, weekly treatment intraperitoneally with a purinergic antagonist suramin resulted in correction of various synaptic abnormalities in multiple pathways. Notably, synaptosome expression of TDP-43 and the key classical complement cascade component C1qa were corrected. Suramin treatment was shown to decrease synaptosomal TDP-43 and C1qa comparatively (27% and 24% respectively) [150]. While investigators hypothesised that disturbances in purinergic signalling may be a common denominator in disease pathogenesis, this study poses the possibility that TDP-43 and C1qa could share common mechanistic pathways and functions at the synapse.

Neurotoxic microglia are shown to promote TDP-43 proteinopathy in PGRN deficiency and the complement cascade may be a mitigating factor. Zhang and colleagues demonstrated that conditioned media from *GRN* knockout microglia is sufficient to promote TDP-43 granule formation, nuclear pore defects, and cell death in excitatory neurons via the complement activation pathway [151]. Consistent with this, deletion of genes encoding key complement components C1qa and C3 mitigated microglial toxicity, rescued TDP43 proteinopathy, and subsequently prevented neurodegeneration [151]. While these findings provide novel insight into the contribution of chronic microglial toxicity to TDP-43 proteinopathy in neurodegeneration, a highlight of this study is the convincing evidence that blocking the activation of a central innate inflammatory pathway (i.e., complement cascade) can mitigate the neurotoxic properties of *GRN* knockout microglia. Thus, this suggests that *GRN* knockout microglia may utilise complement-mediated assembly of protein complexes to promote TDP-43 proteinopathy, indicating an important dynamic may exist between resident innate immune glia, the central innate immune complement pathway, and TDP-43.

There is increasing evidence demonstrating functional links between TDP-43 and various central innate immune inflammatory pathways as outlined above. In addition, multiple studies discussed above have highlighted the potential therapeutic and pharmacological targets that these innate immune inflammatory pathways could provide in the search for disease modifying therapies in ALS and FTD. However, considerable investigation is required into the underlying mechanisms that can explain such functional interactions. Furthermore, investigation that can determine the contribution of such interactions to the pathogenesis of inflammation-driven neurodegeneration in TDP-43 proteinopathies.

## 7. TDP-43 and Adaptive Immunity

GWAS studies report an enrichment of FTD-associated genetic variants in multiple autoimmune disorders [47]. In a recent study by Li et al. 2021 an explicit genetic correlation between ALS and autoimmune diseases was reported. Epidemiological studies from US cohorts have further supported an altered immune system in ALS and FTD that may be exclusively linked to TDP-43. Notably, an increased risk of autoimmune disorders was reported in patients with TDP-43 associated variants of FTD. In one study, patients with semantic FTD or with *GRN* mutations had the highest prevalence of autoimmunity and exhibited increased levels of TNFα, representing a unique pattern of systemic inflammation [152]. In a similar study, the prevalence of non-thyroid autoimmune disorders was reported to be higher in FTD patients with the *C9orf72* repeat expansion, and in patients with coinciding FTD/ALS [153]. In both studies, the increased prevalence of autoimmune diseases clustered around arthritic, cutaneous, and gastrointestinal conditions. However, contrary to these findings, *C9orf72* expansion carriers in a large Finnish cohort study of FTLD patients showed the lowest prevalence of immunological diseases. While still suggesting a role of *C9orf72* in immunoregulation, this study did not provide support for the specific association between TDP-43 pathophysiology and autoimmunity [154]. These differences between US and Finnish FTD cohorts could be attributed to variations in populations or selection bias when allocating patients to groups. Ultimately, epidemiological cohort studies such as these require larger numbers of genetically and pathologically confirmed patients and further research targeted at determining the molecular mechanisms underlying a relationship between autoimmunity, *C9orf72*, and FTD/ALS is required.

As discussed earlier, *C9orf72* expansion has been the focus of multiple rodent model studies demonstrating that loss or elimination of *C9orf72* function profoundly disturbs immune homeostasis and predisposes to autoimmunity, therefore implicating *C9orf72* in the regulation of autoimmunity. *C9orf72* forms a heterodimer with SMCR8 and ablation of SMCR8 in mouse models results in splenomegaly and autoimmune phenotypes like those observed in mice with *C9orf72* deficiency [155]. *C9ORF72* and SMCR8 have interdependent functions in suppressing autoimmunity and negatively regulating lysosomal exocytosis [84]. Collectively, this could explain the increase in the prevalence of autoimmune disease in FTD and ALS *C9orf72* repeat expansions carriers. In addition to *C9orf72*, *GRN* mutations have also been linked to autoimmunity with multiple studies reporting prominent upregulation of serum progranulin levels in patients with various autoimmune diseases. *PGRN* has been identified as a key player in multiple individual autoimmune diseases and antibodies to *PGRN* have been detected in patients with histories of autoimmune conditions [53,54,156,157].

Collectively these studies provide further evidence for immune dysregulation in ALS and FTD, specifically altered adaptive immunity that is intrinsically linked to TDP-43 pathophysiology. The mechanisms underlying the overlap between altered adaptive immunity and TDP-43 proteinopathy warrants further investigation, raising the question as to whether adaptive immune dysregulation could provide a novel target for identifying individuals who may be at risk of developing ALS and FTD or may provide an opportunistic target for future therapies that can manipulate adaptive immune pathways, thus potentially altering the trajectory of neurodegenerative processes.

## 8. Presence of TDP-43 in Other Acute and Chronic Neuroinflammatory Conditions

Neuroinflammation is a pathological feature of various neurological conditions. TDP-43 proteinopathy is observed in non-neurodegenerative conditions including strokes and TBI in addition to being a feature of traumatic injury related-neurodegeneration in CTE. To further elucidate the link between TDP-43 and immune-mediated pathways, the following discussion explores the presence of TDP-43 pathology in acute and chronic inflammatory conditions not primarily driven by neurodegeneration, but rather involving ischemia and brain trauma.

### 8.1. TDP43 and Stroke

Neuroinflammation plays a key role in the pathogenesis of a stroke, with secondary neuroinflammation post stroke promoting further injury and cell death. Conversely however, neuroinflammation is also shown to be beneficial in promoting recovery in strokes as reviewed elsewhere [158]. In a histopathological study performed on human brain tissue biopsies from a panel of anoxic, ischemic, and neoplastic lesions, Lee and colleagues reported an absence of TDP-43 inclusions in ischemic stroke in addition to anoxia and neoplasms, indicating that ischemia does not result in aberrant mislocalisation or accumulation of TDP-43 [159]. However, more recently rodent models of acute ischemic stroke, transient middle cerebral artery occlusion, and subarachnoid haemorrhage (SAH) have consistently shown biochemical and histopathological alterations in TDP-43 following a stroke [160,161,162]. The size of full length TDP-43 (43 kDa) has been shown to decrease in contrast to the size of the 25 kDa C-terminal fragment, which increased after a stroke, explained by the proteolytic cleavage of TDP-43 [160]. In addition, cytoplasmic redistribution, altered nuclear distribution of TDP-43, and an age-related increase in the formation of ubiquitinated TDP-43 after a stroke were also observed.

The dysregulation of TDP-43 expression has been associated with increased microglial activation and innate immune signalling [161]. However unlike in neurodegenerative diseases, abnormal phosphorylation and insolubilisation of TDP-43 and TDP-43 cytoplasmic intracellular inclusions were not observed [160,161,162]. The increase and/or overexpression of cytoplasmic TDP-43 was shown to drive the pathogenic NF-κβ response resulting in increased production of proinflammatory markers, ischemic injury, and increased susceptibility of neurons after stroke, also in an age-dependent manner [161]. The expression of TDP-43 within the CSF and brain tissue of humans has been assessed, with marked elevations in expression of TDP-43 observed in the CSF and brain tissue from patients with SAH relative to healthy controls [162]. SAH enhanced the expression of TDP-43 in the brain of experimental rodents and human subjects and with an increase of TDP-43 occurring in both neurons and glia. Given that inflammation has been proposed as a crucial factor in mediating upregulation and translocation of TDP-43 in pathological conditions, inflammation induced by SAH was suggested to account for the increase in TDP-43 in the CSF of SAH patients [162]. Interestingly, in neurons with cytoplasmic TDP-43 redistribution, ubiquitin was expressed in the cytoplasm with cytoplasmic TDP-43 immunoreactive granules colocalising with ubiquitin granules. However, ubiquitinated intracellular inclusions, a pathological hallmark of TDP-43 proteinopathy, were not observed in rat ischemic brains, alike that of previous rodent models of stroke [160,161].

Finally in a proteomics assessment of the insoluble aggregated proteome following cerebral ischemia in a mouse model of middle cerebral artery occlusion, ischemia/reperfusion induced the aggregation of RNA binding and heat shock proteins with roles in DNA/RNA processing, stress response, and cell signalling. The largest group of aggregated proteins in ischemia was RNA binding proteins including TDP-43 [163]. This suggests a significant molecular overlap between neurodegeneration in ALS and FTD and ischemic stroke and provides evidence that protein aggregation also occurs in acute neuronal injury induced by cerebral ischemia. Collectively these findings indicate that both ischemia and neuronal injury have common characteristics with respect to altered subcellular localisation of TDP-43 [164]. TDP-43 may be specifically ubiquitinated after acute ischemic stroke and structural alterations in TDP-43 are likely dependent on the insult and its location and duration, given phosphorylated TDP-43 was not observed in several rodent stroke models or human stroke patient samples. Moreover, the increase in cytoplasmic TDP-43 with aging could serve as an age-related mediator of inflammation and neuronal injury, with the potential for therapeutic targeting of cytoplasmic TDP-43 post stroke that could modulate post-ischemic inflammation and protect damaged neurons in the ischemic microenvironment [161].

### 8.2. TDP-43, Traumatic Brain Injury and Chronic Traumatic Encephalopathy

TBI results in the activation of multiple inflammatory pathways [165] and is proposed as a risk factor for neurodegeneration. There is increasing evidence to suggest a significant link between brain trauma and TDP-43, with multiple clinical studies and preclinical models of TBI, both single and repetitive, consistently exhibiting TDP-43 proteinopathy including its cleavage, phosphorylation, mislocalisation, and cytoplasmic aggregation [166,167,168,169,170,171,172]. Furthermore, there is evidence to suggest that TDP-43 has a potentially pathogenic role following brain injury as reviewed in detail elsewhere [173].

Given that inflammation is demonstrated to induce cytoplasmic TDP-43 translocation [111], it has been proposed that the enhanced astrocyte and leukocyte response observed in mutant TDP-43 mice following TBI could further increase the burden of phosphorylated TDP-43 granules in neurons, thus further promoting neurodegeneration [172]. Furthermore, TDP-43 proteinopathy has been hypothesised to propagate across neuronal networks [174], therefore brain trauma initiated ALS pathobiochemistry could act as the seed for disease initiation [172]. Supporting this, in ALS mice expressing TDP-43, a model of a mild stab injury to the motor cortex was used to assess the effects on the formation of *p*-TDP-43 cytoplasmic granules. A single stab injury induced the formation of cytoplasmic TDP-43 granules in wild type animals, which peaked at 3 days post injury (dpi) and began declining by 7 dpi, however a much longer response was observed in mutant TDP-43 mice, who continued to accumulate *p*-TDP-43 cytoplasmic granules up until 7 dpi. Moreover, the glial and inflammatory response to TBI was significantly more pronounced in TDP-43 transgenic mice, specifically microglial activation was markedly increased at 3 dpi in TDP-43 mutant mice compared to wild type mice [172]. This suggests there may exist a heightened early or immediate innate immune response that is specific to TDP-43 and that mutant TDP-43 may enhance the neuroinflammatory response to trauma. While microglial activation was markedly increased in the mild stab injury model, in a separate rat model of TBI, TDP-43 proteolysis was associated with astrocyte reactivity. Levels of the 25 kD and 35 kDa fragments of TDP-43 were increased whereas full length TDP-43 (43 kDa) was decreased following TBI. These alterations were associated with neuronal loss and motor impairment and following TBI TDP-43 cleavage products were colocalised with GFAP in reactive astrocytes. Therefore, TBI may induce TDP-43 proteolysis in astrocytes as part of astrocyte activation and downstream functional consequences of TBI [170]. The authors suggested that the early management of TDP-43 proteolysis and its cleavage products in astrocytes could provide a therapeutic target for motor dysfunction following TBI [170].

TBI is linked with the development of CTE, which is characterised by progressive neurodegeneration associated with repetitive head trauma. TDP-43 pathology is present within post-mortem brain tissue in some cases of military related TBI, in sporting athletes who develop CTE and in repetitive head trauma animal models [166,168,173]. The expression of TDP-43 in CTE mimics the pattern of expression seen in FTD-TDP [166] and the strong association of CTE with repetitive TBI in addition to the presence of TDP-43 pathology could represent a specific association between subconcussive brain trauma and TDP-43 pathology [166]. However, it remains unclear how repetitive head trauma causes TDP-43 proteinopathy and neurodegeneration, particularly in the absence of disease-causative mutations.

Research using *Drosophila* models to investigate the link between repetitive brain trauma and ALS show that *Drosophila* have implicated disruptions to altered protein clearance, autophagy pathways, and nucleocytoplasmic transport [175,176]. In *Drosophila* expressing *C9orf72*, repetitive brain trauma showed increased mortality and locomotor dysfunction [175], which may be explained by TBI altering protein clearance pathways such as the ubiquitin-proteosome system or autophagy pathway. This also indicates that TBI could be sufficient to exacerbate the phenotypes associated with ALS-causing genes, in this case *C9orf72*. In the same study, Anderson and colleagues demonstrated the deposition of ubiquitin, p62/*SQSTM1*, TDP-43, and stress granule formation within the *Drosophila* brain following repetitive trauma and, interestingly, *Drosophila* with repeated brain trauma exhibited highly similar pathology to that observed in ALS patients and ALS mouse models [175]. Noteworthy, *SQSTM1*/p62 is an autophagy receptor and has been identified as a rare genetic variant in both ALS and FTD in addition to being able to activate the NF-κβ pathway [72]. In a further follow up study by Anderson and colleagues, proteomics analysis of *Drosophila* brains following repetitive trauma demonstrated that repetitive TBI upregulated nuclear pore proteins, altered nucleoporin stability, nucleocytoplasmic transport proteins, and nucleocytoplasmic transport itself [176]. Interestingly, this upregulation led to TDP-43 mislocalisation, aggregation, and phosphorylation in addition to decreased motor function and lifespan. Furthermore, pathology of nuclear pore glycoprotein 62 (NUP62), an essential component of the nuclear pore complex was observed, with NUP62 pathology colocalising with TDP-43. This study suggests that defects in nucleocytoplasmic transport are associated with traumatic injury, which may mediate TDP-43 pathology. These defects may underly the pathogenesis in CTE, with the NLS -harbouring region of TDP-43 at the centre of this association.

While various studies have investigated TDP-43 proteinopathy across CNS inflammatory disorders outside of neurodegeneration (i.e., stroke and TBI), further research is warranted to investigate the precise mechanisms underlying the highlighted associations to fully explore the paradigm between TDP-43 proteinopathy and acute or chronic CNS insults such as cerebral ischemia and brain trauma. It is important to appreciate that much can be learned from researching TDP-43 proteinopathy in the context of diseases outside of the typical focus of neurodegenerative diseases, material that could be used to further understand TDP-43’s role within the CNS and inform therapeutic targets and pathways that could be manipulated in the context of ALS and FTD.

## 9. Conclusions

**Figure 2:**
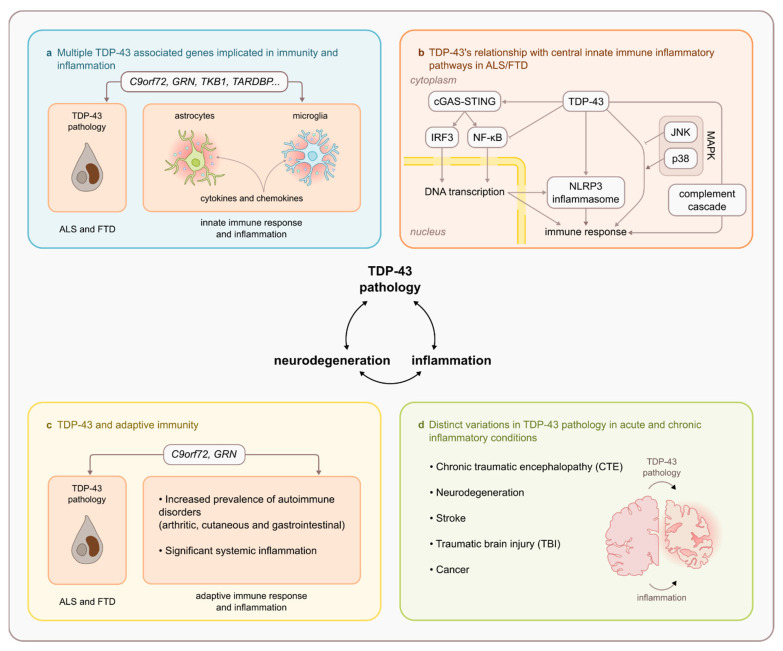
Four key lines of evidence for the specific relationship between TDP-43, immunity, and inflammation within the CNS. (**a**) Multiple ALS and FTD causative and susceptibility genes associated with TDP-43 pathology are directly implicated in immune and inflammatory pathways, notably *C9orf72*, *GRN*, and *TBK1*; (**b**) demonstrated relationship between TDP-43 and central innate immune inflammatory pathways in ALS and FTD including NF-κβ/p65, cGAS/STING, NLRP3 inflammasome, MAPK/JNK/p38, and the complement cascade; (**c**) altered adaptive immunity that is intrinsically linked to TDP-43 pathophysiology, specifically in FTD and ALS *C9orf72* repeat expansion and *GRN* mutation carriers; (**d**) the presence and role of TDP-43 pathology in other acute and chronic inflammatory CNS conditions including stroke, TBI, CTE, and cancer (not discussed in this review). Taken together this evidence points towards a robust and specific relationship between TDP-43, immunity, and inflammation that may provide a platform for the identification of novel immune-mediated targets associated with TDP-43 to aid in discovery and generation of disease-modifying therapies for ALS and FTD.

This review established four key lines of evidence for the specific relationship between TDP-43, immunity, and inflammation (Figure 2). This evidence includes,

1. The involvement of various ALS and FTD causative and susceptibility genes (notably *C9orf72*, *GRN*, and *TBK1*) in immunity and inflammation.
2. A demonstrated relationship between TDP-43 and central innate immune inflammatory pathways including NF-κβ/p65, cGAS/STING, NLRP3 inflammasome, MAPK/JNK/p38, and the innate immune complement cascade.
3. Altered adaptive immunity in ALS and FTD that is intrinsically linked to TDP-43 pathophysiology (notably in *C9orf72* repeat expansion and *GRN* mutation carriers)
4. TDP-43 proteinopathy is observed in other acute and chronic inflammatory CNS conditions (notably stroke, TBI and CTE).

This review has also identified evidence that warrants further investigation including:

- Investigation into the substantial amount of evidence supporting TDP-43’s relationship with immunity and inflammation that centres around microglia.
- Determining the manner and involvement of TDP-43 structural and functional sites (e.g., NLS and RRM1 domain) with key inflammatory pathways (e.g., NF-κβ/p65)
- Investigation of the mechanisms underlying TDP-43’s role in triggering cytoplasmic mitochondrial DNA release and activating central inflammatory pathways (i.e., cGAS/STING)
- Deciphering the association between TDP-43 and autoimmunity to determine whether systemic inflammation is a risk factor for TDP-43 proteinopathy or the possibility of common shared mechanisms between TDP-43 proteinopathies and adaptive immune dysregulation.
- Explore the mechanisms underlying TDP-43’s presence and involvement in strokes, TBI, and CTE to determine whether shared mechanisms exist in the context of neuroinflammation.

In summary, inflammatory pathways and immune-mediated mechanisms present a promising opportunity to explore therapeutic manipulation and biomarkers of inflammation that can inform disease progression during life in ALS and FTD patients expressing TDP-43 proteinopathy. Furthermore, investigation of the role of TDP-43 in other CNS disorders that also exhibit neuroinflammation may provide novel avenues to uncover the mechanisms underlying TDP-43 proteinopathy in the CNS.

## Figures and Tables

**Table 1:** Published evidence implicating ALS and FTD causative and susceptibility genes associated with TDP-43 in key immune and inflammatory pathways.

Gene	Protein	Relationship to TDP-43	Associated Diseases	Published Evidence
*TARDBP*	TDP-43	Gene encoding for TDP-43 protein	ALS, FTD rare variant	Gliosis in transgenic mice Increased phagocytosis in microglia Mediates non-cell autonomous neurotoxic effects Intrinsic dysregulation of microglia induced by TDP-43 depletion > triggers abnormal synapse loss [48,49]
*C9orf72*	Guanine nucleotide exchange *C9orf72*	TDP-43 is pathological feature of C9orf72 expansion in ALS and FTD	ALS, FTD, AD	C9orf72 is extensively linked to neuroinflammation and microglial activation Increased presence of C9orf72 in dendritic immune cells and microglia C9orf72 can activate NLRP3 inflammation C9orf72 knockout mice exhibit a systemic proinflammatory state, resulting in severe autoimmunity [50,51,52]
*GRN*	Progranulin	TDP-43 is pathological feature of GRN mutation in FTD	FTLD-GRN, CLN11 disease	PGRN promotes lysosomal dysfunction and production of complement cascade components preferentially affecting synaptic connections Upregulation of immune system and complement cascade genes in GRN knockout mice GRN knockout mice with decreased PGRN leads to excessive accumulation of activated microglia and increased secretion of proinflammatory cytokines PGRN suppresses neuroinflammation after acute focal cerebral ischemia [53,54]
*TBK1*	Serine/threonine-protein kinase TBK1	3rd most common genetic cause of FTLD-TDP [46]	ALS, FTD rare variant	Encodes protein kinase with an established role in the regulation of the immune response, autophagy, and inflammation [55,56]
*UBQLN2*	Ubiquilin-2	UBQLN2 dysregulation in neurons can drive NF-κβ activation and cytosolic TDP-43 aggregation [57]	FTD rare variant	Drives NF-κβ activity and cytosolic TDP-43 aggregation in neuronal cells ALS-linked mutations in the UBQLN2 gene found to be associated with dysfunction of autophagy, neuroinflammation, and formation of stress granules [57,58,59]
*ATXN2*	Ataxin-2	Link between *ATXN2* and TDP-43 proteinopathy established [60]	ALS, Parkinson’s disease (late onset), Spinocerebellar ataxia type 2 (SCA2)	Defined interconnected pathways including innate immunity, complement system, lysosome, and phagosome pathways [61]
*TREM2*	Triggering receptor expressed on myeloid cells 2	Reported novel interaction between TREM2 and TDP-43	Susceptibility gene FTD	Immune and/or inflammatory gene upregulated in aged microglia Promotes microglial activation, survival, chemotaxis, and phagocytosis Increased expression after brain injury and stroke [62,63,64]
*TMEM106B*	Transmembrane protein 106B	Common variants in TMEM106B serve as a distinct risk factor for TDP-43 pathology in older individuals without FTLD [65]	Susceptibility gene FTD	TMEM106B/GRN dysfunction and TDP-43 pathology results in increased expression of anti-inflammatory microglial genes in the frontal cortex of protective allele carriers [66]
*OPTN*	Optineurin	Optineurin inclusions detected in small subset ALS and FTD with TDP pathology [67]	Glaucoma, ALS with or without FTD rare variant	Diminished inflammation after bacterial infection in OPTN knockout mice Reduced expression of cytokine interferon beta (IFNβ) secretion in macrophages from knockout mice and OPTN transgenic mice [68,69,70]
*SQSTM1*	Sequestosome-1, p62	Sequestration of SQTSM1 into TDP-43 aggregates, leads to inhibition of proteasome function and autophagy and promotes the accumulation of toxic, misfolded proteins [71]	Paget disease of bone 3, FTD/ALS rare variant	Activates NF-κβ Increased expression of p62 in activated peripheral macrophages [72,73]
*VCP*	Transitional endoplasmic reticulum ATPase	Major component of ubiquitinated inclusions of FTLD with VCP mutation is TDP-43 [74]	FTD	Increased inflammatory cytokines in mutation-positive patients Mediates degradation of the NF-κβ inhibitor IκB kinase after cytokine treatment Activation of NLRP3 inflammasome in myoblasts from mutation-positive patients Increased activation of macrophages in VCP- mutant transgenic mice [75,76]
*CYLD*	Ubiquitin carboxyl-terminal hydrolase CYLD	CYLD directly interacts with TBK1, OPTN, and p62 [77]	ALS, FTD	Suppresses NF-κβ activation Role in immune system regulation and response to infectious disease [78,79]
